# Kiwifruit *SVP2* gene prevents premature budbreak during dormancy

**DOI:** 10.1093/jxb/erx014

**Published:** 2017-02-03

**Authors:** Rongmei Wu, Tianchi Wang, Ben A W Warren, Andrew C Allan, Richard C Macknight, Erika Varkonyi-Gasic

**Affiliations:** 1 The New Zealand Institute for Plant & Food Research Limited (Plant & Food Research) Mt Albert, Private Bag 92169, Auckland Mail Centre, Auckland, New Zealand; 2 School of Biological Sciences, University of Auckland, Private Bag 92019, Auckland, New Zealand; 3 Department of Biochemistry, University of Otago, Dunedin, New Zealand

**Keywords:** ABA, *Actinidia*, budbreak, bud dormancy, dehydration, kiwifruit, SVP, transcriptome

## Abstract

MADS-box genes similar to *Arabidopsis thaliana SHORT VEGETATIVE PHASE* (*SVP*) have been implicated in regulation of flowering in annual species and winter dormancy in perennial species. However, the underlying regulatory mechanisms remain to be identified. In this study, the role of kiwifruit *SVP2* was explored using ectopic transgenic expression in kiwifruit species with different chilling requirements and the model species tobacco, followed by transcriptomic analysis of transgenic kiwifruit plants. Ectopic expression of *SVP2* affected the duration of dormancy in a high-chill kiwifruit *Actinidia deliciosa*. This effect could be overcome by sufficient winter chilling. *SVP2* had a minimal effect on the duration of dormancy in a low-chill kiwifruit *A. eriantha*. Expression in a tobacco cultivar with photoperiodic regulation of flowering resulted in retarded vegetative growth but no impact on flowering. Transcriptomic analyses of the kiwifruit *SVP2* transgenic and control lines identified 92 significantly differentially expressed genes potentially involved in *SVP2*-mediated growth repression during dormancy, suggesting a role complementary to abscisic acid (ABA). This study has demonstrated that kiwifruit *SVP2* has an integrative role in suppression of meristem activity to prevent precocious budbreak before the fulfilment of winter chilling requirements.

## Teaser

Overexpression of *SVP2* in kiwifruit delays budbreak before sufficient winter chilling. *SVP2*-mediated vegetative growth restriction involves stress response pathways, and commonalities exist between Arabidopsis and kiwifruit SVP targets.

## Introduction

In temperate horticultural woody perennials, winter dormancy is of particular importance both to avoid unfavourable winter conditions, and to synchronize budbreak and flowering in the following spring (Cooke *et al.*, 2012; Yamane, 2014). Winter dormancy is a dynamic process, defined as a period between bud set in the autumn and budbreak in the spring, when no visible growth occurs. Dormancy has been divided into para-, endo-, and eco-dormancy phases (Lang *et al.*, 1987). Para-dormancy is the suspension of growth caused by factors outside the meristem but within the plant, such as apical dominance. Endo-dormancy is the deepest state of dormancy, when budbreak is prevented by endogenous factors specific to the meristem, which stop the growth even under favourable external conditions. Eco-dormancy is when the growth capacity is restored in the meristem, but remains suspended because of unfavourable external environmental factors and can be released when conditions become permissive. A certain amount of chilling in a bud is often required for the transition from endo-dormancy to eco-dormancy (Lang *et al.*, 1987; Rohde *et al.*, 2007).

Bud development can be dissected into bud formation, acclimation to dehydration and cold, and dormancy. Each of these steps is associated with specific sets of regulatory and marker genes and metabolites (Ruttink *et al.*, 2007). Recent studies of metabolites and gene expression reconstruct the temporal sequence of events during bud development. At least three main regulatory programmes control the onset of dormancy, namely signal perception, hormone alteration, and transcription factors (Shim *et al.*, 2014). Similarly, multiple functional categories of differentially expressed genes (DEGs) have been identified during dormancy release, including stress response, sugar metabolism, hormone response, cell cycle and DNA processing, energy generation, transcription factors, and signal transduction (Fabbroni, 2009). This has been further reinforced by a number of independent studies (Walton *et al.*, 2009; Leida *et al.*, 2012; Liu *et al.*, 2012; Nishitani *et al.*, 2012; Bai *et al.*, 2013; da Silveira Falavigna *et al.*, 2013; Ueno *et al.*, 2013; Zhong *et al.*, 2013; Howe *et al.*, 2015). However, genetic regulation of dormancy remains largely unknown.

The first suggestion that MADS-box genes might be important regulators of dormancy came from a study of the peach (*Prunus persica*) *evergrowing* (*evg*) mutant. Deletion of six tandem arrayed *DORMANCY-ASSOCIATED MADS-BOX* (*DAM*) genes in peach resulted in a complete lack of dormancy under cold or short-day (SD) induction, while the expression of a subset of these genes was elevated during endo-dormancy (Bielenberg *et al.*, 2008; Li *et al.*, 2009; Yamane *et al.*, 2011). Similarly, a negative correlation of expression with endo-dormancy release was observed for six tandem arrayed *DAM* genes predicted to act as transcriptional repressors in Japanese apricot (*Prunus mume*) (Yamane *et al.*, 2008; Sasaki *et al.*, 2011). Ectopic expression of one of these genes in transgenic poplar resulted in premature growth cessation and terminal bud set, demonstrating a role in growth inhibition in the model woody perennial plant (Sasaki *et al.*, 2011). Genes encoding homologs of DAM transcription factors are differentially regulated during dormancy in many horticultural woody perennials (Mazzitelli *et al.*, 2007; Bielenberg *et al.*, 2008; Diaz-Riquelme *et al.*, 2009; Li *et al.*, 2010; Ubi *et al.*, 2010; Sasaki *et al.*, 2011; Yamane *et al.*, 2011; Liu *et al.*, 2012; Bai *et al.*, 2013; da Silveira Falavigna *et al.*, 2013; Mimida *et al.*, 2015; Porto *et al.*, 2016), suggesting a conserved role in dormancy, and a major quantitative trait locus (QTL) for chilling requirement and bloom date overlapped the peach genomic regions where *DAM* genes are located (Zhebentyayeva *et al.*, 2014). However, the underlying mechanism and mode of action remain poorly understood and the genetic evidence from diverse species is limited.

DAM proteins are closely related to *Arabidopsis thaliana* flowering time regulators SHORT VEGETATIVE PHASE (SVP) and AGAMOUS-LIKE 24 (AGL24). Arabidopsis SVP and AGL24 are central regulators in the flowering regulatory network, with high sequence similarity but opposite functions. Their mode of action includes interaction with other proteins, resulting in either repressing or activating complexes that regulate floral transition and maintain floral meristem identity (Michaels *et al.*, 2003; Gregis *et al.*, 2006; Lee *et al.*, 2007, 2014; Liu *et al.*, 2007, 2009; Li *et al.*, 2008), or direct binding to the CArG motifs in floral activators, such as *FLOWERING LOCUS T* (*FT*) and *SUPPRESSOR OF OVEREXPRESSION OF CONSTANS 1* (*SOC1*) (Lee *et al.*, 2007; Posé *et al.*, 2013). In a herbaceous perennial leafy spurge (*Euphorbia esula*), *DAM*-like and *FT*-like genes are reciprocally and differentially expressed during winter dormancy transition, implying a similar mechanism in regulation of dormancy (Horvath, 2009).

Woody perennials usually have multiple *SVP* homologues, resulting from lineage- and species-specific expansions within the *SVP*/*AGL24* MADS-box subfamilies (Wells *et al.*, 2015). Four *SVP* genes have been identified in the kiwifruit species *Actinidia chinensis* and *A. deliciosa*, with differential ability to delay flowering in Arabidopsis. Expression of *SVP1*, *SVP2*, and *SVP4* was elevated in kiwifruit buds over the winter dormancy period, and the relative transcript abundance was higher in colder regions, suggesting roles in bud dormancy and flowering (Wu *et al.*, 2012). In contrast, *SVP3* accumulation in buds did not demonstrate seasonal changes, but ectopic expression caused abnormal flower development, reduced petal pigmentation, and abnormal fruit and seed development, supporting a role in repression of reproductive development (Wu *et al.*, 2014). To understand the mechanism of *SVP*-mediated regulation of kiwifruit bud dormancy, budbreak, and flowering, *SVP2* was ectopically expressed in a high chilling requirement species *A. deliciosa*, a low chilling requirement species *A. eriantha*, and in *Nicotiana tabacum* ‘Maryland Mammoth’. Detailed physiological and transcriptomic analyses of *35S*:*SVP2 A. deliciosa* transgenic lines were performed.

## Materials and methods

### Plant transformation and growth conditions

*SVP2* coding sequence under the control of the *Cauliflower mosaic virus* (CaMV) *35S* promoter (Wu *et al.*, 2012) was transformed into *Agrobacterium tumefaciens* strain EHA105 for transformation into kiwifruit *A.* deliciosa ‘Hayward’ [*A. deliciosa* (A. Chev.) C.F. Liang et A.R. Ferguson, also referred to as *A. chinensis* var. *deliciosa* (A.Chev.) A. Chev.] and *A. eriantha* Benth. The same construct was transformed into *A. tumefaciens* strain GV3101 for transformation into tobacco (*N. tabacum* ‘Maryland Mammoth’). A reporter gene *uidA* (*GUS*) under the control of the CaMV *35S* promoter (*35S:GUS*) in appropriate *Agrobacterium* strains was used to transform control plants. The transformation procedure for *A. deliciosa* was previously described (Wang *et al.*, 2006, 2007). Transformation of *A. eriantha* was according to a previously described protocol (Wang *et al.*, 2006, 2007), with modifications to media composition. The regeneration medium contained half-strength Murashige and Skoog (1/2 MS) agar medium (Murashige and Skoog, 1962), 2 mg l^−1^ 6-benzylaminopurine (BAP), 1 mg l^−1^ zeatin, 0.2 mg l^−1^ indole-3-butyric acid (IBA), 300 mg l^−1^ timentin, and 150 mg l^−1^ kanamycin. The shoot elongation medium contained 1/2 MS, 0.1 mg l^−1^ zeatin, 0.5 mg l^−1^ IBA, 300 mg l^−1^ timentin, and 50 mg l^−1^ kanamycin. Once their roots were established, transgenic plants were transferred to soil and grown in a containment glasshouse for 18 months at Plant & Food Research, Auckland, New Zealand. Budbreak time and flowering time for transgenic *A. deliciosa* and *A. eriantha* were assessed in the following spring season. *Nicotiana tabacum* transformation was carried out on young leaf discs excised from *in vitro* grown shoots (Horsch *et al.*, 1985). Transgenic tobacco plants were grown in a containment glasshouse at 20 °C under SD conditions (8/16 h light/dark). The seeds from these transgenic plants were collected and germinated on 1/2 MS agar medium (Murashige and Skoog, 1962) supplemented with 50 µg ml^−1^ kanamycin. Following the segregation tests, two homozygous lines were chosen and six T_2_ generation plants of each line were used for detailed analysis.

For clonal propagation, *A. deliciosa 35S:SVP2* Line 1 young shoots were collected and surface sterilized using 25% bleach (containing 1.25% sodium hypochlorite) for 20 min, followed by rinsing with sterile water five times. The nodes with axillary buds were excised and transplanted to MS medium. New shoots initiated from these axillary buds, and subsequently seven clonal plants were generated. Once roots were established, plants were transferred to ambient containment glasshouse conditions over 18 months. To initiate dormancy, plants were maintained in SD conditions for 6 weeks (18 °C, 14 h dark and 10 h light intensity at 300–600 µmol s^−1^ m^−2^) and subsequently subjected to 4 weeks of fluctuating temperature conditions (14–20 °C during the day and 4–10 °C at night) with an average 9.5 h day length (maximum light intensity at 1000–2000 µmol s^−1^ m^−2^). After 100% leaf drop, lateral buds were collected and the plants were subjected to chilling at 3–7 °C for up to 8 weeks.

### *Determination of* A. deliciosa *dormancy status*

Dormancy status was determined as described previously (Voogd *et al.*, 2015). Briefly, stem cuttings with a single lateral bud were excised on a regular basis from each plant, the lower ends were immersed in water and maintained at budbreak forcing conditions (20 °C, 14 h photoperiod of white light and 70–80% humidity), and the number of days until visible budbreak was recorded. A minimum of three cuttings for each plant were used.

### RNA extraction and expression studies

Total RNA was extracted from kiwifruit tissue as previously described (Chang *et al.*, 1993). Total RNA was isolated from tobacco leaf using the Trizol reagent (Invitrogen). A 5 μg aliquot of total RNA was treated with DNase I (Ambion) and reverse transcribed at 37 °C using the BluePrint^®^ Reagent kit for reverse transcription–PCR (RT–PCR) (TaKaRa) according to the manufacturer’s instructions. Amplification and quantification were carried out using the LightCycler^®^ 480 System and SYBR Green I Master Mix (Roche Diagnostics). Reactions were performed in quadruplicate, and a non-template control was included in each run. Thermal cycling conditions were 95 °C for 5 min, followed by 50 cycles of 95 °C for 10 s, 60 °C for 10 s, and 72 °C for 20 s, followed by a melting temperature cycle, with constant fluorescence data acquisition from 65 °C to 95 °C. The data were analysed using the ratio of target to reference and calculated with the LightCycler^®^480 software 1.5 (Roche Diagnostics). The expression was normalized to previously characterized reference genes, kiwifruit *Actin* (Wu *et al.*, 2012) and tobacco *Ntα-Tub1* (Pattanaik *et al.*, 2010). Primer sequences used in this study are listed in Supplementary Table S1 at *JXB* online.

### RNA-seq library construction and sequencing

Bud samples were collected from *A. deliciosa SVP2* transgenic and control plants on 28 June 2013, 14 August 2013, and 4 October 2013. Three biological replicates from independent overexpressing *SVP2* transgenic and control lines were used, with 8–10 lateral buds per replicate at each stage. Total RNA was extracted from lateral buds as previously described (Chang *et al.*, 1993). RNA samples were treated with RNase-free DNase I (Life Technologies, New Zealand) followed by an RNA cleanup kit (Zymo Research). RNA integrity was measured using the RNA 6000 Nano kit and the 2100 Bioanalyzer instrument (Agilent). The library preparation and sequencing were performed by Macrogen, Korea, using Illumina HiSeq™ 2000. Raw sequence data in fastq format were filtered to remove the adaptors and the low-quality reads, leaving a total of 328 million clean reads for subsequent analysis.

### De novo *transcriptome assembly*

The single-end forward reads from each library were initially mapped to the kiwifruit *A. chinensis* ‘Hongyang’ reference genome (Huang *et al.*, 2013) using Bowtie2 with default settings (Langmead and Salzberg, 2012). The average mapping rates were low, at ~50% (Supplementary Table S2), reflecting actual sequence differences between the two closely related *Actinidia* species, as well as the poorly predicted and annotated gene models of the draft reference genome. To increase mapping rates, *de novo A. deliciosa* transcriptome assembly was carried out using the short-read assembly program, Trinity version (0.0.1) (http://trinityrnaseq.sourceforge.net/). Transcriptome assembly completeness was assessed using cegma_v2.4.010312 (http://korflab.ucdavis.edu/datasets/cegm​a/). False and duplicated contigs were removed using EvidentialGene VERSION 2013.07.27 (http://arthropods.eugenes.org/Evidential​Gene/). This Transcriptome Shotgun Assembly project has been deposited at DDBJ/EMBL/GenBank under the accession no. GEYI00000000. A total of 68 454 unique contigs were assembled and annotated using reciprocal BLAST alignment to TAIR 10 Arabidopsis protein databases (https://www.arabidopsis.org). To enrich the reference transcript library, we combined *de novo* assembled contigs with the *A. deliciosa* EST library containing 6454 ESTs (Crowhurst *et al.*, 2008) as the reference transcriptome database. The single-end forward reads from each library were mapped to this reference using Bowtie2 (Langmead and Salzberg, 2012). The average mapping rates increased to 75% (Supplementary Table S2) and this set was used for subsequent identification of DEGs. Principal component analysis (PCA) (Stacklies *et al.*, 2007) was performed using the DESeq v2.10 package.

### Analysis of differentially expressed genes

The DEGs between each sample set were detected with DESeq v2.10 (Anders and Huber, 2010). The cut off of *Padj*<0.05 value, followed by the absolute value of logFC (log2 fold change) of not less than 1.0 were considered as significantly differentially expressed genes. The gene expression unit was calculated using the RPKM method (reads per kilobase of transcript per million mapped reads). Annotations were obtained by BLAST of amino acid sequence to Arabidopsis amino acid sequences. Hierarchical clustering as well as heatmap analysis of DEGs were described in McAtee (2014). The best Arabidopsis (TAIR 10) hit was used for Gene Ontology (GO) term classification, and significance was established by singular enrichment analysis (SEA) coupled with available background data of Arabidopsis [false discovery rate (FDR) ≤0.05], using AgriGO Version1.2 (Du *et al.*, 2010). The abiotic stress response and hormone response were established using the Arabidopsis eFP Browser (http://bbc.botany.utoronto.ca/efp/cgi-bin/efpWeb.cgi) (Winter *et al.*, 2007).

## Results

### *Overexpression of* SVP2 *delays budbreak in a high-chill kiwifruit* A. deliciosa

**Fig. 1.**
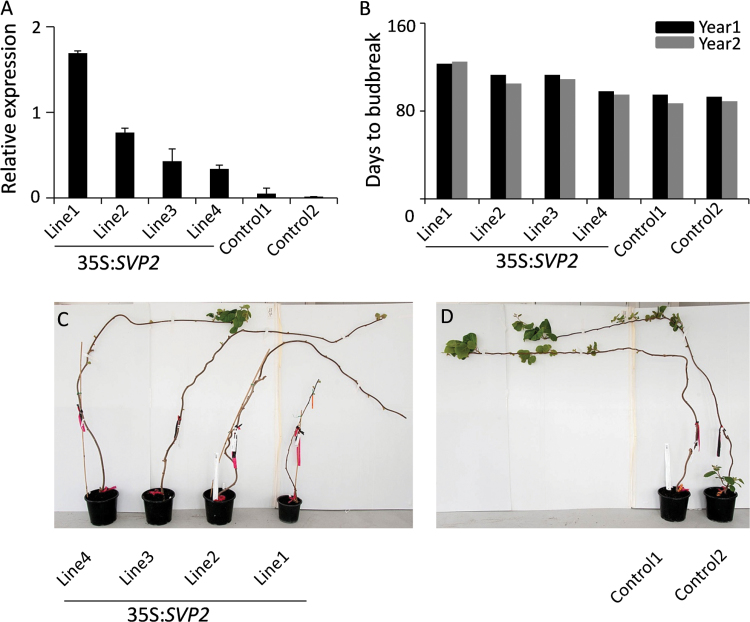
Constitutive expression of *SVP2* delays budbreak in *Actinidia deliciosa*. (A) Relative expression of *SVP2* in four *35S:SVP2* transgenic plants and two control plants. The expression was normalized to kiwifruit *Actin.* Error bars represent the SE of four replicate reactions. (B) Days to budbreak after 100% leaf drop in late autumn. The first visible leaf in spring was recorded as budbreak. (C, D) Transgenic *A. deliciosa* plants and control plants in the middle of spring.

To evaluate the role of *SVP2* gene in kiwifruit, transgenic *A. deliciosa* lines with *SVP2* cDNA driven by the CaMV *35S* promoter were generated, using the standard transformation protocol (Wang *et al.*, 2012) and the CaMV *35S* promoter-driven *uidA* (*GUS*) construct as control. *Actinidia deliciosa* has a high chilling requirement, long dormancy, and late spring budbreak. Normal adventitious shoot formation and growth were observed with the control construct, but the initiation of adventitious buds and shoot elongation were impaired when the *SVP2* construct was used. After multiple transformation experiments, four independent transgenic lines were obtained, with varying levels of *SVP2* transgene expression (Fig. 1A). No difference in autumn growth cessation, leaf drop, timing of bud-set, and bud formation could be detected between any of the *SVP2* transgenic and control lines over the period of 2 years, but a significant delay in the first visible budbreak during the spring season in both years was observed for *SVP2* lines. This delay correlated with levels of *SVP2* transgene expression and was most prominent in Line 1 (Fig. 1B, C). This line showed slow and weak growth and remained significantly smaller than the control lines over a period of 2 years (Fig. 1C, D).

### *Cold treatment of* SVP2 A. deliciosa *transgenic lines*

**Fig. 2.**
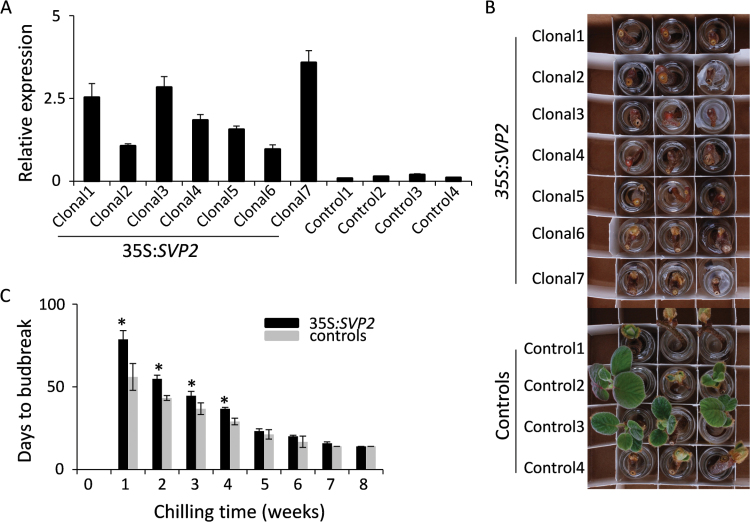
Chilling alleviates *SVP2*-mediated repression of budbreak. (A) Relative expression of *SVP2* in clonal transgenic and control kiwifruit plants. The expression was normalized to kiwifruit *Actin.* Error bars represent the SE of four replicate reactions. (B) Delayed budbreak of cuttings from *SVP2* transgenic plants after 3 weeks of chilling accumulation. (C) Days to budbreak were calculated as the average date of budbreak of seven clonal transgenic and four control plants. Black and grey bars denote transgenic and control plants, respectively. Error bars represent the SE of budbreak time of seven transgenic and four control plants. Asterisks indicate significantly delayed budbreak time of *SVP2* plants versus controls (*P*<0.05; Student’s *t*-test).

In glasshouse conditions, *A. deliciosa* plants perceived insufficient chilling because of mild winters in Auckland, New Zealand. To address how chilling conditions related to the *SVP2*-mediated delay of budbreak, we selected *SVP2* Line 1 with the highest transgene expression for further analysis. Seven clonal plants were generated from Line 1, which all expressed the *SVP2* transgene (Fig. 2A). The plants were allowed to grow in the containment glasshouse for a period of 18 months, before dormancy was induced by conditions mimicking autumn and early winter. Four control plants were subjected to the same treatment. Dormant plants were exposed to cold treatment for 8 weeks, and three single node cuttings were collected weekly to evaluate dormancy status (Fig. 2B). Average budbreak time demonstrated negative correlation with the duration of chilling (Fig. 2C). Without chilling, no budbreak was observed in cuttings taken from either control or *SVP2* plants. In *SVP2* plants subjected to cold, a significantly delayed budbreak compared with control plants was detected for up to 4 weeks of cold treatment. A delay of 23, 12, and 8 d was recorded for plants treated for 1, 2, and 3 weeks, respectively. After 4 weeks, average budbreak was still delayed by 8 d, but this delay decreased gradually after 5 weeks of cold treatment to no detectable difference after 8 weeks of cold treatment (Fig. 2C).

### *Overexpression of* SVP2 *in a low-chill kiwifruit* A. eriantha

**Fig. 3.**
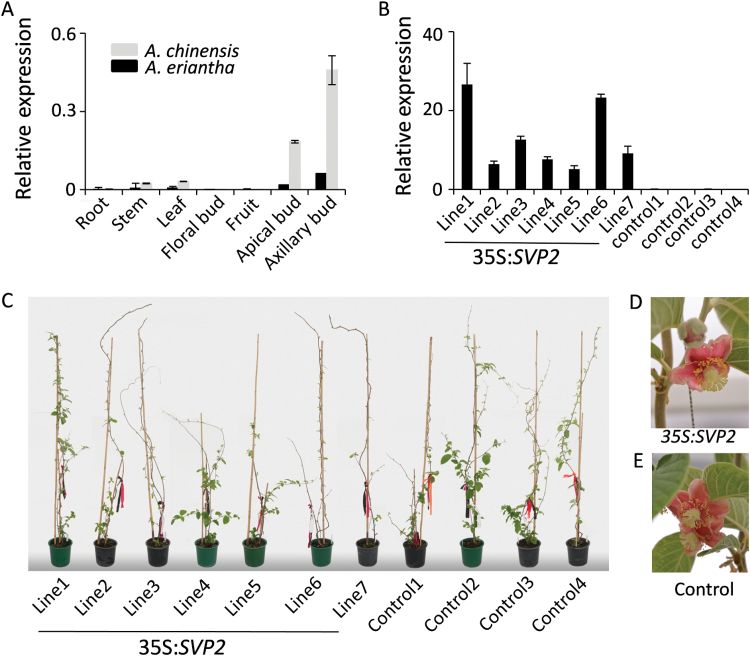
Constitutive expression of *SVP2* in a low-chill kiwifruit *Actinidia eriantha*. (A) Relative expression of *SVP2* in *A. eriantha* and *A. chinensis* tissues. The level of expression was normalized to *Actin*. Error bars represent SEs for three replicate reactions. (B) Relative expression of *SVP2* in seven *35S:SVP2* transgenic plants and four control plants. The expression was normalized to kiwifruit *Actin.* Error bars represent the SE of four replicate reactions. (C) Transgenic *A. eriantha* plants and control plants in early spring. (D, E) Transgenic and control *A. eriantha* flowers.

The restored budbreak timing of sufficiently chilled *35S*:*SVP2* transgenic *A. deliciosa* prompted us to study the role of *SVP2* in a kiwifruit which has a low chilling requirement, *A. eriantha*. In this kiwifruit species, *SVP* gene sequences and expression are highly comparable and therefore likely to be functionally conserved as previously described in *A. chinensis* and *A. deliciosa* (Wu *et al*., 2012, 2014) (Fig. 3A). An additional advantage of *A. eriantha* is the fast reproductive maturity and prolific flowering in glasshouse conditions (Wang *et al.*, 2006), facilitating the study of the role of *SVP2* in reproductive onset and development. Initial attempts at regeneration and transformation using standard protocols optimized for *A. eriantha* (Wang *et al.*, 2006) were unsuccessful. Transformation with the control construct resulted in normal callus formation, initiation of adventitious buds, and subsequent growth, but browning and aborted shoot tip development were recorded with the *SVP2* construct (Supplementary Fig. S1). Similar results were obtained in an attempt to transform another kiwifruit species, *A. chinensis*, suggesting that overexpression of *SVP2* plays a detrimental role in regeneration and growth. In an attempt to alleviate transgene-mediated growth restriction, the media were modified to reduce salt concentration (Han *et al.*, 2010) and to change the balance of plant growth regulators, eventually resulting in efficient regeneration, increased initiation of adventitious buds, and improved bud survival. Seven transgenic lines with moderate to high levels of *SVP2* transgene expression and four control lines were generated (Fig. 3B) and monitored for budbreak and flowering. A slight delay in the first visible budbreak in some *SVP2* transgenic lines was recorded, but no clear correlation could be made between the transgene expression levels and timing of budbreak (Fig. 3C; Table 1). The flowering time and number of flowers was highly variable (Table 1), but all lines produced flowers with normal morphology and pigmentation (Fig. 3D, E).

**Table 1.** *Phenotypic analysis of 35S:SVP2 transgenic* Actinidia eriantha Days to budbreak were recorded as days from 100% leaf drop to the first visible budbreak. Number of breaking buds was recorded as the total number of developing shoots at the end of spring. Days to flowering were recorded as days from the first visible budbreak to the appearance of the first floral bud. Number of flowers was counted as total flowers per line

Transgenic lines	Days to budbreak	Number of breaking buds	Days to flowering	Number of flowers
Line 1	40	28	18	11
Line 2	43	21	18	11
Line 3	45	23	14	3
Line 4	40	25	18	5
Line 5	40	20	25	5
Line 6	45	23	25	7
Line 7	43	22	25	35
Control 1	40	32	nil	0
Control 2	40	34	32	1
Control 3	40	34	25	3
Control 4	40	20	18	22

### *Overexpression of* SVP2 *affects plant growth and seed germination, but not flowering time and petal colour in transgenic tobacco*

**Fig. 4.**
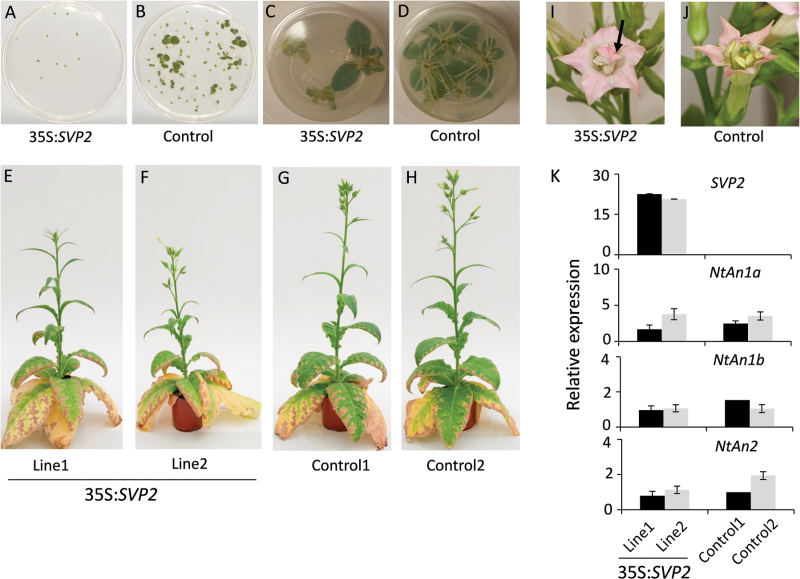
Constitutive expression of *SVP2* affects vegetative development in transgenic tobacco ‘Maryland Mammoth’. (A, B) Seed germination of *35S:SVP2* plants compared with control plants, 25 d after seeds stratification on MS plates. (C, D) Slow root formation in transgenic *35S:SVP2* plants compared with control plants. (E–H) Two lines of transgenic *35S:SVP2* plants compared with control plants under SD conditions. (I, J) Mutant transgenic *SVP2* flower compared with control. The arrow indicates the petaloid stamen in the transgenic flower. (K) Relative expression of *NtAn1a*, *NtAn1b*, *NtAN2*, and the *SVP2* transgene in petals of transgenic plants compared with control plants. Black and grey bars represent relative expression of two independent lines. The expression of each gene was normalized to tobacco *Ntα-Tub1.* Error bars represent the SEs for four replicate reactions.

Less efficient transformation in all tested kiwifruit species and delayed budbreak in *A. deliciosa* suggested that *SVP2* played a detrimental role in vegetative growth that could be overcome by changes in growth conditions (chilling and modified media composition), while not affecting flower development and petal colour, in contrast to reports for kiwifruit gene *SVP3* (Wu *et al.*, 2014). To investigate whether kiwifruit *SVP2* had a conserved growth-restriction effect and further evaluate a role in flowering time and reproductive development, *SVP2* cDNA driven by the CaMV *35S* promoter was transformed into an SD flowering tobacco variety ‘Maryland Mammoth’. The progeny of two independent transgenic tobacco lines were subjected to detailed analysis. Seed germination and root growth were delayed compared with those in controls (Fig. 4A–D; Table 2). Significant differences were observed in the height of transgenic *SVP2* plants, although the plant architecture and secondary growth were visually similar (Fig. 4E–H; Table 2). Flowering time and the number of flowers produced were similar, but more sterile flowers were found on *SVP2* plants than on controls (Table 2). Occasional homeotic conversion of stamen to petal was observed (Fig. 4I, J); however, the petal pigmentation and expression of the tobacco anthocyanin regulators, bHLH genes, *NtAn1a* and *NtAn1b*, and R2R3 MYB, *NtAN2* (Pattanaik *et al.*, 2010; Bai *et al.*, 2011) were comparable with those in control lines (Fig. 4K).

**Table 2.** Phenotypic analysis of 35S:SVP2 transgenic tobacco Data are presented as means and the SE of six individuals for each lines. Days for seed germination were recorded as days from sterilization to visible germination on MS plates. Total leaf number was counted when the first floral bud was visible. Plant height was expressed as centimetres when the first visible floral bud appeared. Total number of flowers and sterile flowers were counted on inflorescences

Transgenic Lines	Seed germination (d)	Total leaf number	Plant height (cm)	Total number of flowers	Number of sterile flowers
Line 1	11 ± 2.3	18.7 ± 0.4	42.5 ± 3.3	26.1 ± 1.2	21.0 ± 2.5
Line 2	13 ± 1.2	20.0 ± 1.2	31.1 ± 1.1	32.5 ± 1.5	27.7 ± 4.1
Control 1	7 ± 0.0	19.3 ± 0.4	56.6 ± 3.6	28.0 ± 2.5	13.5 ± 1.6
Control 2	7 ± 0.0	22.3 ± 0.4	49.0 ± 2.1	34.0 ± 2.5	14.5 ± 0.5

### *Overexpression of* SVP2 *in* A. deliciosa *leads to transcriptomic changes during winter dormancy*

To understand the molecular mechanisms underlying *SVP2*-mediated growth repression in kiwifruit, a transcriptome analysis of *SVP2* transgenic lines (t) and control lines (c) at different dormancy stages was performed. Sampling times corresponded to (i) the endo-dormant stage following 100% leaf drop; (ii) transition to eco-dormancy after exposure to winter temperature; and (iii) initiation of budbreak. The dormancy status of bud samples was confirmed by single node cutting assays. Budbreak was delayed in *SVP2* plants at the first two time points, and *SVP2* plants remained mostly endo-dormant at the first time point (Supplementary Fig. S2). No difference in budbreak time was detected between *SVP2* and control lines at the third sampling point. For that reason, only *SVP2* transgenic plant bud samples at this time point were analysed further. A total of 15 libraries were prepared and 60–70 million RNA-seq reads were generated for each library. PCA of these RNA-seq reads demonstrated clear separation between sampling dates, but less variation between *SVP2* transgenic and control lines at corresponding sampling dates. The sample set at time point 3 was more variable, reflecting transcriptomic changes at an advanced developmental phase just before visible budbreak (Supplementary Fig. S2).

**Fig. 5.**
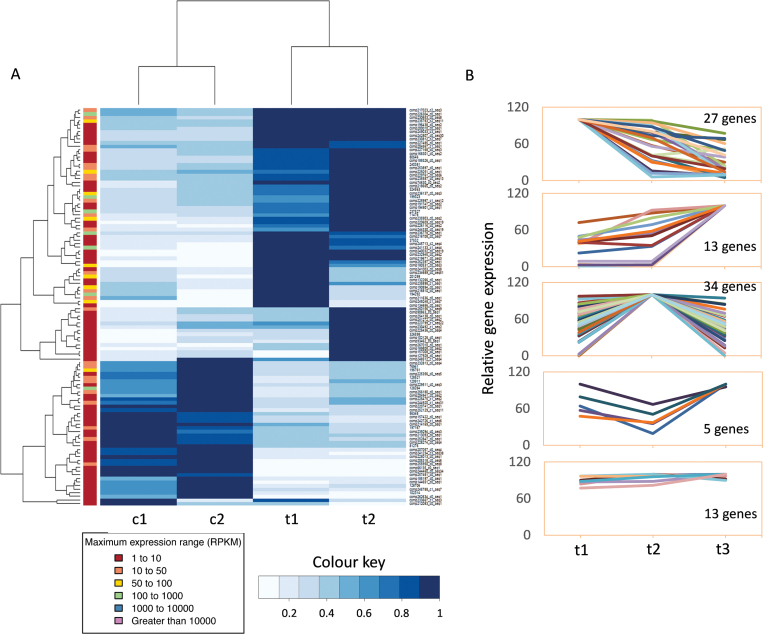
*SVP2*-mediated transcriptomic changes during winter dormancy. (A) Hierarchical clustering of 92 differentially expressed genes identified by transcriptome analysis of *SVP2* transgenic lines (t) and control lines (c) at the endo-dormant stage (1) and transition to eco-dormancy after exposure to winter temperature (2). The columns represent comparisons between samples, and rows represent individual genes. The gene name, gene symbol, and RPKM of each gene can be found in Supplementary Table S5 and the higher resolution image is provided as Supplementary Fig. S4. The colour chart of blue and white indicates the RPKM value. Blue and white represent increased and decreased gene expression, respectively. (B) Transcriptome analysis of *SVP2* transgenic lines during endo-dormancy (t1), eco-dormancy (t2), and initiation of budbreak (t3) identified five expression patterns for 92 differentially expressed genes.

Comparison of *SVP2* transgenic and control plant transcriptomes identified 253 genes significantly differentially expressed in the buds collected at the first time point (t1-c1) (Supplementary Table S3), and 226 in the buds collected at the second time point (t2-c2) (Supplementary Table S4), with 92 in the common set, 54 and 38 consistently up- and down-regulated, respectively, in *SVP2* transgenic plants (Fig. 5A; Supplementary Table S5). Annotation of the closest Arabidopsis homologue found that 76 DEGs in t1-c1 and 41 DEGs in t2-c2 have been previously identified as direct Arabidopsis SVP targets (Tao *et al.*, 2012; Gregis *et al.*, 2013), while 69 DEGs in t1-c1 and 58 DEGs in t2-c2 have been associated with dormancy in poplar, leafy spurge, and kiwifruit (Horvath *et al.*, 2008; Walton *et al.*, 2009; Howe *et al.*, 2015). Functional classification using GO enrichment analysis identified several categories of biological processes that were significantly affected in the *SVP2* transgenic plants, most notably stress response in the t1-c1 and t2-c2 sets. The molecular functions of catalytic and transferase activity were significantly enriched in the common gene set (Supplementary Table S6). Further interrogation of the closest Arabidopsis homologue expression data available through the eFP browser (Winter *et al.*, 2007) revealed that a large proportion of identified genes responded to abiotic stress or plant hormone treatments, most commonly osmotic and cold stress and abscisic acid (ABA) treatment. Of the 92 common genes at both time points, 31 were homologous to Arabidopsis genes that are ABA or osmotic/drought responsive (Supplementary Table S5).

Analysis of *SVP2* plant transcriptomes across different dormancy stages identified five types of expression patterns for the common set of 92 DEGs over the three time points (t1, t2, and t3); increasingly up- or down-regulated from endo-dormancy to budbreak, transiently up- or down-regulated during progression to eco-dormancy, and expressed to a similar level over the dormancy period (Fig. 5B). The accuracy and reproducibility of the transcriptome analysis results was confirmed by real-time RT–PCR analysis of a subset of candidate genes (Supplementary Fig. S3).

## Discussion

### SVP2 *delays shoot outgrowth but may not be sufficient for the onset of dormancy in kiwifruit*

In many woody perennials, *SVP* genes have been associated with winter dormancy. In particular, *DAM* genes from *Prunus persica* and *P. mume* have been advanced as key regulators of winter dormancy (Bielenberg *et al.*, 2008; Jiménez *et al.*, 2010; Sasaki *et al.*, 2011; Yamane *et al.*, 2011). As these *Prunus* spp. are recalcitrant to transformation, Sasaki *et al.* (2011) demonstrated a potential role by heterologous expression of *Prunus DAM6* in poplar. Overexpression of kiwifruit *SVP2* in kiwifruit provides a system to ratify the growth inhibitory function of *SVP* genes by ectopic expression in the species of origin. The low regeneration efficiency suggested that overexpression of *SVP*-like genes strongly inhibited outgrowth of plants in tissue culture, potentially explaining the absence of reports on the role of these genes in the species from which they were isolated.

In natural conditions, onset of kiwifruit bud dormancy can be induced by autumn SD and cooler/fluctuating temperature conditions (Brundell, 1976; Lionakis and Schwabe, 1984). The *SVP2* kiwifruit lines exhibited no difference in the morphology of the shoot apex and axillary bud formation in comparison with control plants. In particular, premature growth termination and early bud set have not been observed in transgenic *A. deliciosa* or *A. eriantha SVP2* lines grown in ambient conditions in summer (long days). The autumn SD and low temperature conditions did not visibly enhance the leaf senescence, leaf drop, and bud set in transgenic lines. Instead, transgenic plants showed a delay in axillary budbreak in the spring, suggesting that *SVP2* was associated with maintenance of deep bud dormancy. This is in contrast to findings reported in transgenic poplar where expression of *Prunus DAM6* resulted in premature growth cessation followed by terminal bud set (Sasaki *et al.*, 2011). A possible explanation is that the onsets of terminal and lateral bud dormancy rely on somewhat different mechanisms. In kiwifruit, the shoot tip aborts instead of forming a terminal bud; abortion is preceded by growth cessation and is initiated by tissue necrosis in the subapical zone (Foster *et al.*, 2007). The timing of shoot tip abortion is negatively correlated with the shoot expansion rate and can occur at any time, resulting in short or long shoots. This high developmental plasticity makes visual observations of growth cessation in kiwifruit difficult; however, evidence from both kiwifruit and tobacco *SVP2* lines confirms a role in growth inhibition. Delayed germination followed by slower root and shoot development all indicate that *SVP2* can act as a growth repressor in tobacco. In addition, both *SVP2* and *Prunus DAM6* performed a role in lateral bud endo-dormancy in kiwifruit and poplar, respectively (Sasaki *et al.*, 2011), as demonstrated by delayed shoot outgrowth. Therefore, *SVP2* in kiwifruit performs as a growth repressor once dormancy has been established, but may not be sufficient to suppress kiwifruit growth in permissive conditions. We therefore propose that *SVP2* has a key role in suppressing meristem activity in dormant axillary buds.

### *Winter chilling can over-ride* SVP2-*mediated growth inhibition in kiwifruit*

Plant dormancy has been divided into three well-defined phases, para-, endo-, and eco-dormancy (Lang *et al.*, 1987). While growth can resume during para- and eco-dormancy, accumulation of chilling is required to release endo-dormancy, to allow budbreak and floral competency in the following spring (Linsley-Noakes and Allan, 1987; Walton *et al.*, 2001; Snelgar *et al.*, 2008). The normal chilling requirement for *A. deliciosa* ‘Hayward’ is ~800 h (Linsley-Noakes and Allan, 1987), after which dormancy is fully alleviated. Insufficient chilling results in delayed budbreak followed by reduced flower and fruit development. Ectopic *SVP2* therefore mimics the effects of insufficient chilling, further delaying budbreak, either by maintenance of deep dormancy or by reduction of shoot outgrowth rate. This effect is gradually reduced and becomes negligible after chilling for the period of ~800 h (5 weeks), suggesting that elevated *SVP2* is not sufficient to suppress growth once adequate chilling requirements are met. This finding is consistent with our observations in the low-chill kiwifruit species *A. eriantha*, where elevated *SVP2* had only a minor effect, strongly suggesting that kiwifruit *SVP2* does not play a role in chilling-mediated dormancy release. Instead, it would appear that *SVP2* prevents premature growth before full chilling is perceived.

Interestingly, elevated expression of *SVP2* in shoot buds during dormancy and its decline prior to budbreak (Wu *et al.*, 2012) suggests transcriptional regulation of SVP2 action. This is consistent with other reports of elevated *DAM* gene expression during dormancy and the suggestions that winter chilling repressed *DAM* gene expression, resulting in dormancy release (Horvath *et al.*, 2008; Yamane *et al.*, 2008; Li *et al.*, 2009). However, the failure of ectopically expressed *SVP2* to maintain dormancy after sufficient chilling indicated additional regulation at the post-transcriptional level. Possible mechanisms are unknown and may include post-transcriptional or post-translational modifications, differential protein stability, or alternative protein–protein interactions. Degradation of SVP protein and differential interactions with other MADS-box protein partners have been established as important during floral transition in Arabidopsis (Michaels *et al.*, 2003; Gregis *et al.*, 2006; Liu *et al.*, 2007, 2009; Lee *et al.*, 2014) and may be instrumental in regulation of dormancy and budbreak in other plant species, including kiwifruit.

### SVP2 *affects vegetative growth but has no obvious effect on reproductive development and petal colour*

Previously, we reported that kiwifruit *SVP2* and *SVP3* had a differential ability to delay flowering in Arabidopsis and rescue the Arabidopsis *svp41* phenotype (Wu *et al.*, 2012). Despite their high sequence similarity, only *SVP3* was capable of delaying flowering and complementing the *svp41* mutant. Conversely, elevated *SVP3* had no obvious effect on vegetative growth, dormancy, or flowering time in transgenic *Actinidia* or tobacco (Wu *et al.*, 2014), consistent with the lack of increased expression in shoot buds during dormancy (Wu *et al.*, 2012). Instead, elevated *SVP3* delayed flower development and reduced petal pigmentation in transgenic *A. eriantha* and tobacco, through interference with transcription of the key anthocyanin pathway regulators (Wu *et al.*, 2014). While *SVP2* had no effect on the timing of flower development and anthocyanin biosynthesis in petals, it was capable of delaying budbreak in the high-chill kiwifruit species and retarding vegetative growth in transgenic tobacco, consistent with elevated expression in shoot buds during dormancy. Therefore, these two closely related kiwifruit *SVP* genes have acquired different roles as growth repressors in kiwifruit. Co-expression of *SVP2* and *SVP3* in the shoot buds suggests that they may require different interacting partners to perform diverse functions. Similarly, the inability of the *SVP2* transgene to prevent growth in permissive conditions and upon sufficient chilling may indicate a requirement for protein complexes, in which other interacting partners respond directly to environmental stimuli (e.g. accumulation of chilling).

### *Transcriptomic analysis indicates that* SVP *genes may mimic the ABA effect*

Transcriptomic changes in *SVP2* transgenic lines revealed 92 putative *SVP2* target genes, significantly up- and down-regulated over two stages of dormancy. Almost half of the genes were typically regulated in response to stress, most often osmotic and cold treatment, with a subset also identified as ABA-responsive genes. These results are consistent with previous findings of coinciding expression of *DAM4–DAM6* and several ABA and drought stress response genes during dormancy in peach cultivars (Leida *et al.*, 2012). ABA is an important growth inhibitor previously associated with dormancy; ABA was elevated during endo-dormancy and dropped following the transition to eco-dormancy in several species (Rinne *et al.*, 1994; Le Bris *et al.*, 1999; Rohde and Bhalerao, 2007; Horvath *et al.*, 2008). Consequently, genes associated with response to ABA are often cold, drought, and stress regulated and preferentially expressed during endo- and eco-dormancy (Horvath *et al.*, 2008). ABA affects dormancy progression through its action on dehydrins or membrane permeability (Campoy *et al.*, 2011). Accordingly, kiwifruit genes identified as differentially expressed in *SVP2* lines often show homology to well-described genes associated with the dehydration process. *Responsive to dehydration 22* (*RD22*) is a molecular link between ABA signalling and abiotic stress, and its expression has been used as a reliable ABA early response marker in many plants (Shinozaki and Yamaguchi-Shinozaki, 2000; Matus *et al.*, 2014). *RD22* has been associated with grape bud dormancy (Mathiason *et al.*, 2009) and Arabidopsis seed dormancy (Yamaguchi-Shinozaki and Shinozaki, 1993). In this current study, two kiwifruit transcripts with homology to *RD22* were highly up-regulated at the dormancy stage, but gradually declined prior to budbreak in transgenic *SVP2* lines. The *Early-Responsive to Dehydration Stress* (*ERD*) genes have been collectively characterized in Arabidopsis as genes that are rapidly induced by dehydration stress. Three transcripts with similarity to an *ERD*-like gene together with a *late embryogenesis abundant protein* (*ATECP31*) were down-regulated in *SVP2* overexpression lines, suggesting that different dehydration pathways existed between transgenic and control lines. Other osmotic- and ABA-responsive genes included *ASPARAGINASE B1*, *GLYOXYLASE 17*, and *Vacuolar processing enzyme*, which were all up regulated in *SVP2* transgenic lines. Predicted *SVP2* targets also include multiple protein kinases and phosphatases, potentially involved in ABA-induced signal transduction, and several transcription factors also associated with stress and ABA.

It is unclear at this stage if ABA metabolism itself is affected by overexpression of *SVP2* in kiwifruit. One of the ABA biosynthesis pathway genes, *NCED3*, was elevated in the *SVP2* transgenic lines in the early stage of dormancy (t1-c1), but not differentially expressed at t2-c2. Similarly, we found no evidence of major differences in ABA concentration between transgenic *SVP2* and control lines in the samples corresponding to those collected for RNA-seq analysis during dormancy (data not shown). Therefore, it is possible that *SVP2* mimics the ABA effect by targeting of genes and pathways associated with the dehydration process. In that case, *SVP2* may be targeting the dehydration response only before sufficient chilling is perceived. After sufficient chilling, this pathway may be disrupted and the presence of *SVP2* becomes insufficient to repress growth. Interestingly, a significant overlap between kiwifruit *SVP2* and Arabidopsis SVP targets was revealed; in particular, in the t1-c1 set, 30% of top Arabidopsis hits have been reported to be directly regulated by SVP (Tao *et al.*, 2012; Gregis *et al.*, 2013), suggesting conservation of the mechanism of action between taxa. However, many of the well-defined Arabidopsis SVP target genes, such as homologues of *FT* and *SOC*, were not affected by elevated *SVP2* expression, consistent with no demonstrated role for *SVP2* in flowering and suggesting that *SVP2* preferentially controls only specific aspects of dormancy.

In summary, this study has demonstrated a growth-inhibiting role for kiwifruit *SVP2*, mediated by ABA and dehydration response pathways, regulating the timing of meristem activity to avoid unfavourable winter conditions and prevent precocious budbreak.

## Supplementary data

Supplementary data are available at *JXB* online.

Fig. S1. Transformation of *Actinidia eriantha*.

Fig. S2. Evaluation of *A. deliciosa* transgenic and control buds at three stages for the RNA-seq experiment.

Fig. S3. qPCR validation of RNA-seq expression profiles.

Fig. S4. High resolution image of hierarchical clustering presented in Fig. 5.

Table S1. qPCR primer sets used in the RNA-seq validation.

Table S2. Summary of RNA-seq experiments.

Table S3. List of the differentially expressed genes (log2 >±1, *Padj*<0.05) between transgenic *SVP2* (t1) and control (c1) in bud samples collected on 28 June 2013.

Table S4. List of the differentially expressed genes (log2 >±1, *Padj*<0.05) between transgenic *SVP2* (t2) and control (c2) in bud samples collected on 14 August 2013.

Table S5. List of the common set of differentially expressed genes in buds collected at both time points (t1-c1 versus t2-c2).

Table S6. List of GO enrichment analysis results (FDR <0.05) for differentially expressed genes in t1-c1, t2-c2, and the common gene set differentially expressed at both time points.

## Supplementary Material

supplementary_figures_S1_S4

supplementary_table_S1

supplementary_table_S2

supplementary_table_S3

supplementary_table_S4

supplementary_table_S5

supplementary_table_S6
