# Supplementary material for: Kiwifruit SVP2 gene prevents premature budbreak during dormancy
Source: J Exp Bot. 2017 Feb 3;68(5):1071–82. doi: 10.1093/jxb/erx014 (PMC5853213; doi:10.1093/jxb/erx014)
Supplement: supplementary_figures_S1_S4 [file erx014_suppl_supplementary_figures_s1_s4.pdf]

Fig. S1

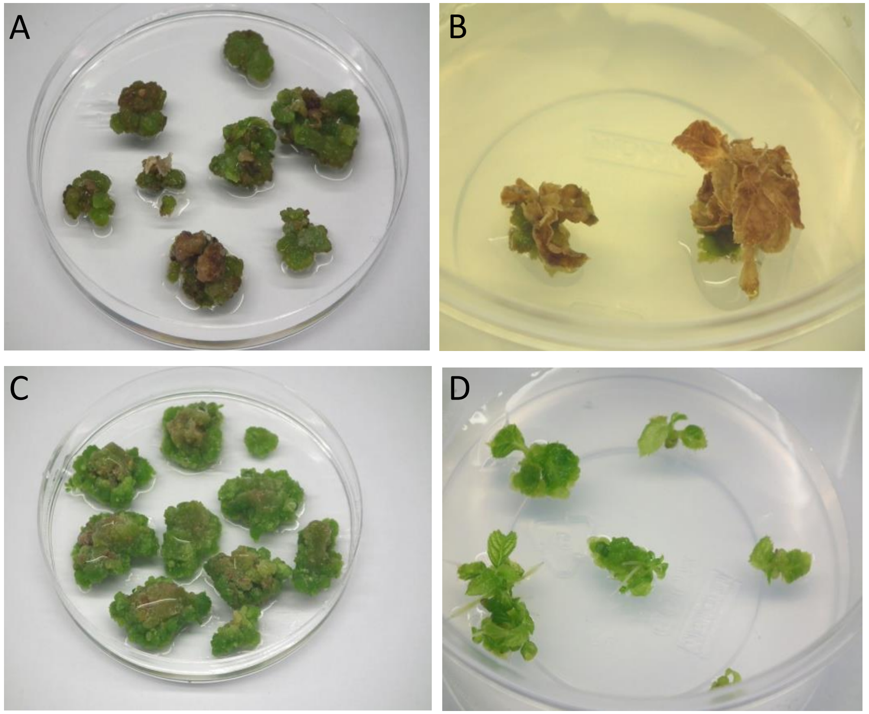

Fig. S1 Transformation of *Actinidia eriantha*. (A) Browning of adventitious *SVP2* shoots on a standard regeneration medium. (B) Shoot tip die-back on a standard elongation medium. (C) Healthy *SVP2* callus with adventitious buds on modified regeneration medium. (D) Healthy transgenic shoots produced on modified elongation medium.

Fig. S2

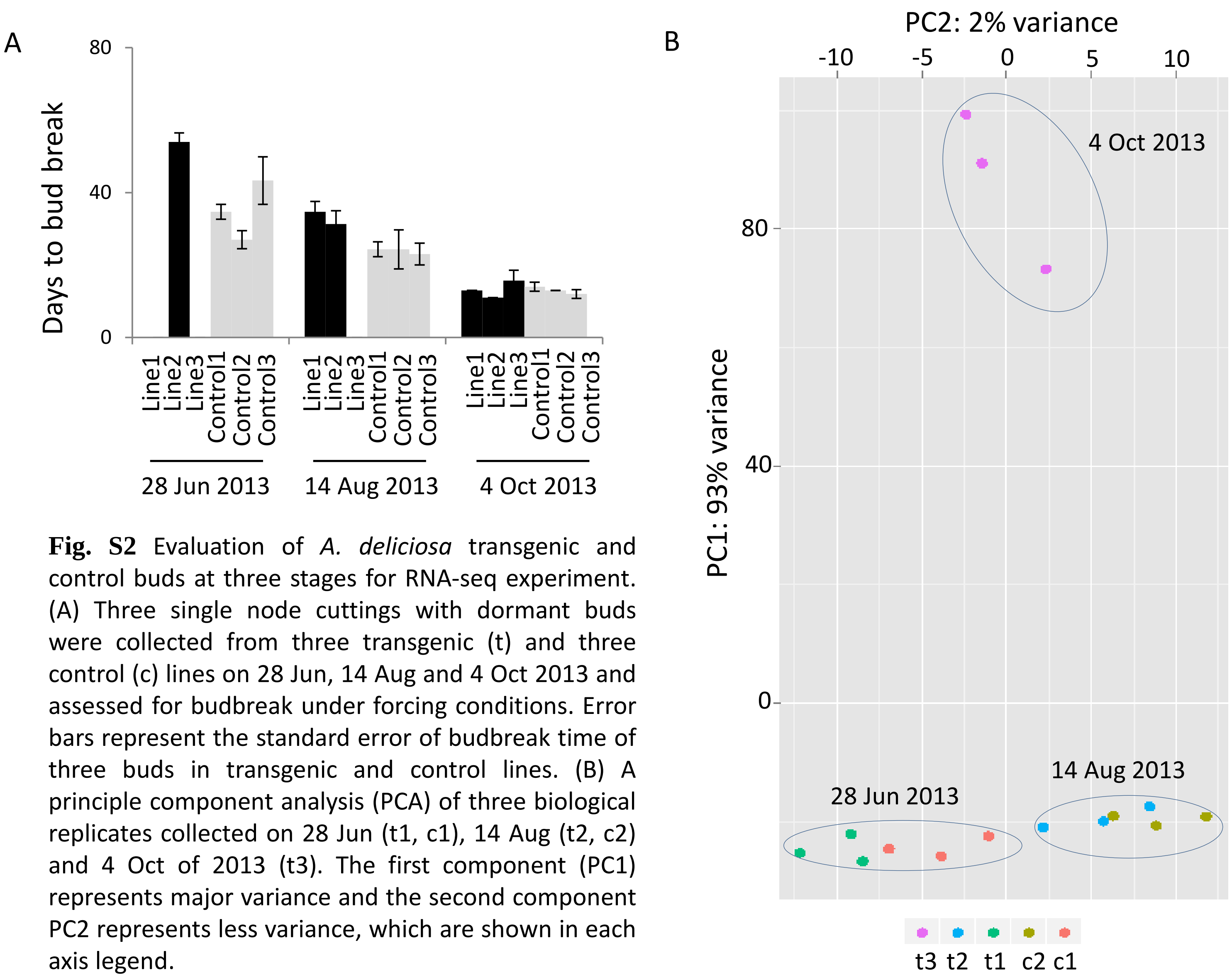

Fig. S2 Evaluation of *A. deliciosa* transgenic and control buds at three stages for RNA-seq experiment. (A) Three single node cuttings with dormant buds were collected from three transgenic (t) and three control (c) lines on 28 Jun, 14 Aug and 4 Oct 2013 and assessed for budbreak under forcing conditions. Error bars represent the standard error of budbreak time of three buds in transgenic and control lines. (B) A principle component analysis (PCA) of three biological replicates collected on 28 Jun (t1, c1), 14 Aug (t2, c2) and 4 Oct of 2013 (t3). The first component (PC1) represents major variance and the second component PC2 represents less variance, which are shown in each axis legend.

Fig. S3

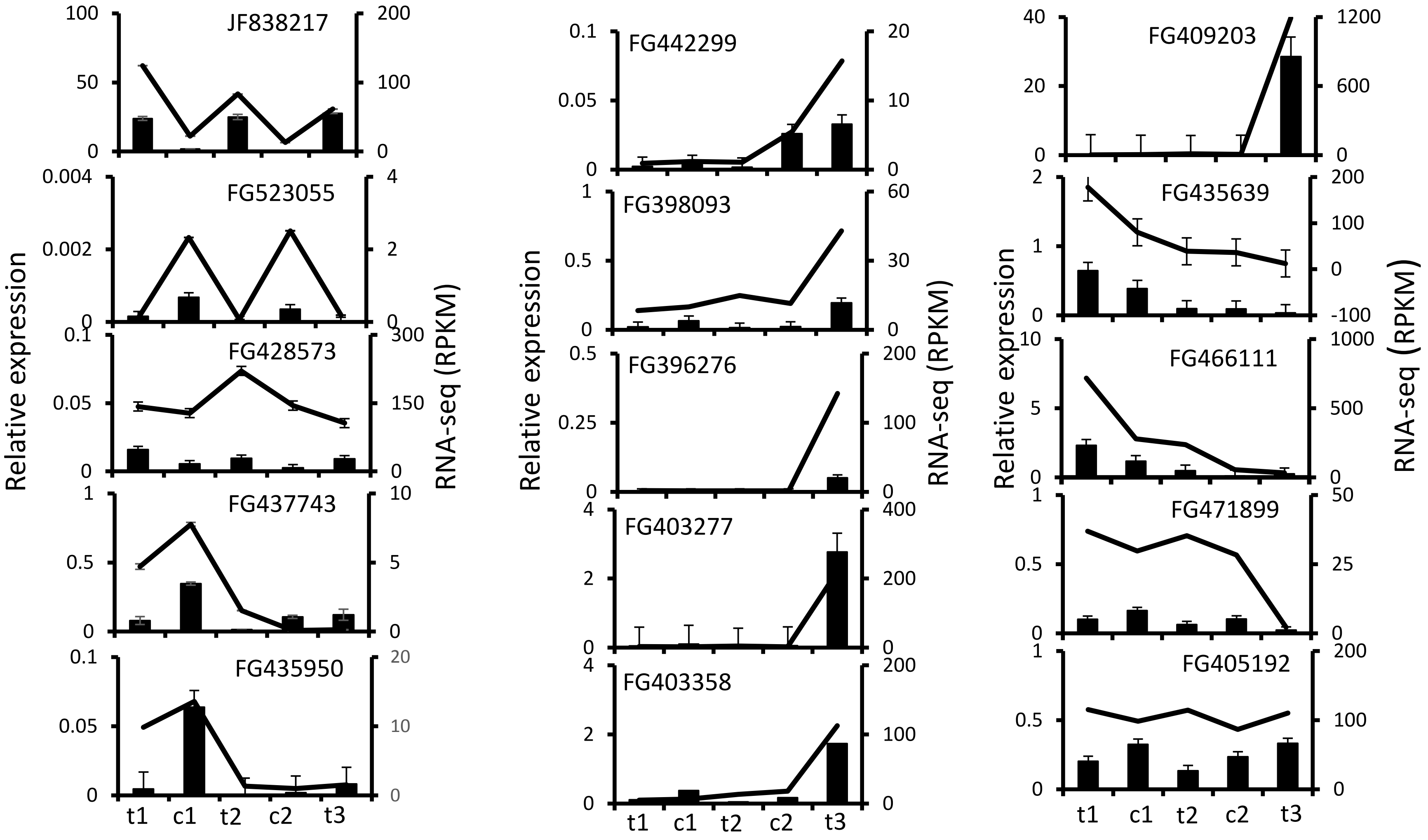

Fig. S3 qPCR validation of RNA-seq expression profiles. 15 genes were randomly selected from differentially expressed genes in c1, c2, t1, t2 and t3. The left y-axis indicates relative gene expression levels determined by qPCR. Error bars represent the standard error for four replicate reactions. The right y-axis indicates gene expression levels calculated by the RPKM method.

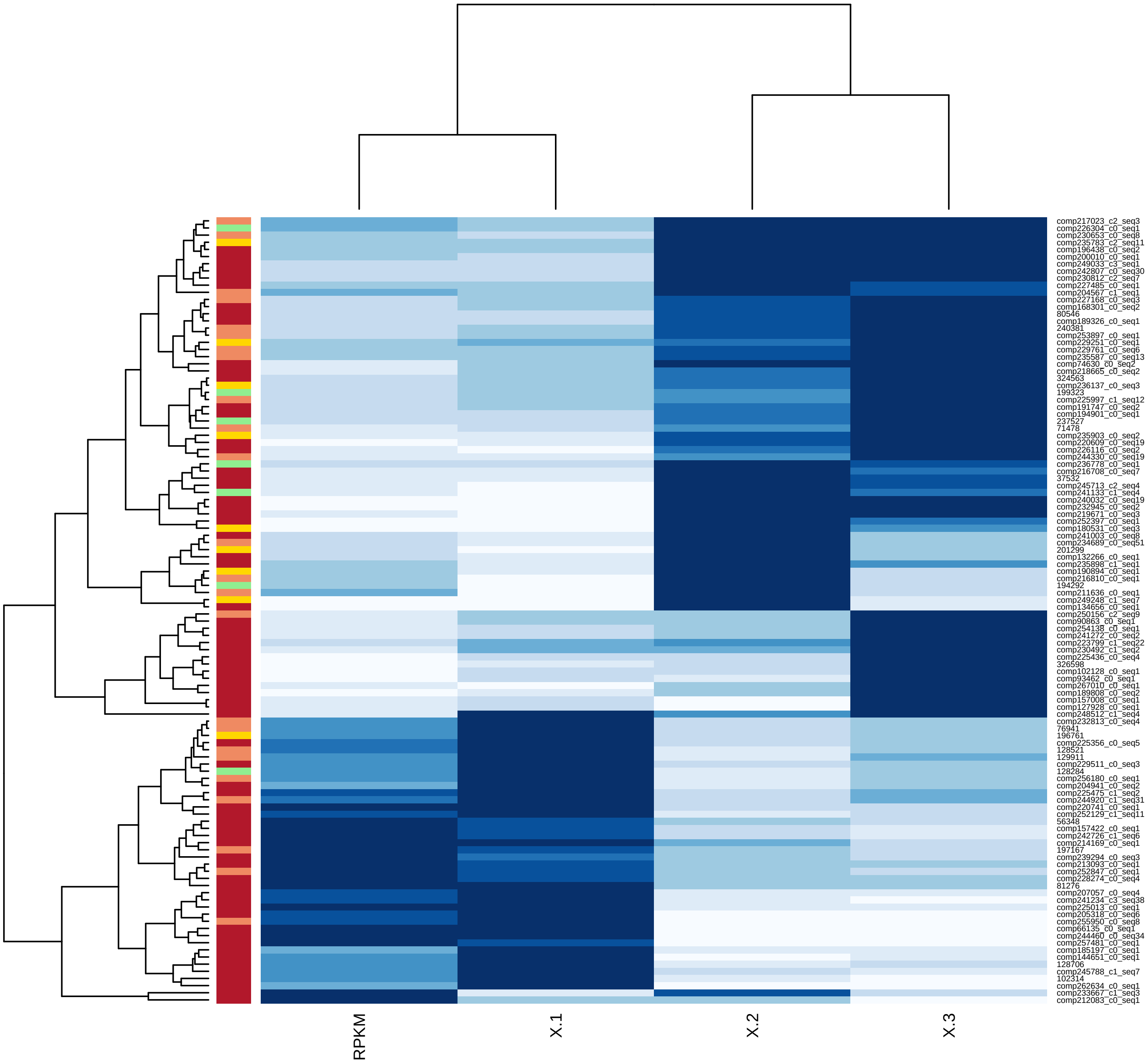

comp217023_c2_seq3
comp226304_c0_seq1
comp230653_c0_seq8
comp235783_c2_seq11
comp196438_c0_seq2
comp200010_c0_seq1
comp249033_c3_seq1
comp242807_c0_seq30
comp230812_c2_seq7
comp227485_c0_seq1
comp204567_c1_seq1
comp227168_c0_seq3
comp168301_c0_seq2
80546
comp189326_c0_seq1
240381
comp253897_c0_seq1
comp229251_c0_seq1
comp229761_c0_seq6
comp235587_c0_seq13
comp74630_c0_seq2
comp218665_c0_seq2
324563
comp236137_c0_seq3
199323
comp225997_c1_seq12
comp191747_c0_seq2
comp194901_c0_seq1
237527
71478
comp235903_c0_seq2
comp220609_c0_seq19
comp226116_c0_seq2
comp244330_c0_seq19
comp236778_c0_seq1
comp216708_c0_seq7
37532
comp245713_c2_seq4
comp241133_c1_seq4
comp240032_c0_seq19
comp232945_c0_seq2
comp219671_c0_seq3
comp252397_c0_seq1
comp180531_c0_seq3
comp241003_c0_seq8
comp234689_c0_seq51
201299
comp132266_c0_seq1
comp235898_c1_seq1
comp190894_c0_seq1
comp216810_c0_seq1
194292
comp211636_c0_seq1
comp249248_c1_seq7
comp134656_c0_seq1
comp250156_c2_seq9
comp90863_c0_seq1
comp254138_c0_seq1
comp241272_c0_seq2
comp223799_c1_seq22
comp230492_c1_seq2
comp225436_c0_seq4
326598
comp102128_c0_seq1
comp93462_c0_seq1
comp267010_c0_seq1
comp189808_c0_seq2
comp157008_c0_seq1
comp127928_c0_seq1
comp248512_c1_seq4
comp232813_c0_seq4
76941
196761
comp225356_c0_seq5
128521
129911
comp229511_c0_seq3
128284
comp256180_c0_seq1
comp204941_c0_seq2
comp225475_c1_seq2
comp244920_c1_seq31
comp220741_c0_seq1
comp252129_c1_seq11
56348
comp157422_c0_seq1
comp242726_c1_seq6
comp214169_c0_seq1
197167
comp239294_c0_seq3
comp213093_c0_seq1
comp252847_c0_seq1
comp228274_c0_seq4
81276
comp207057_c0_seq4
comp241234_c3_seq38
comp225013_c0_seq1
comp205318_c0_seq6
comp255950_c0_seq8
comp66135_c0_seq1
comp244460_c0_seq34
comp257481_c0_seq1
comp185197_c0_seq1
comp144651_c0_seq1
128706
comp245788_c1_seq7
102314
comp262634_c0_seq1
comp233667_c1_seq3
comp212083_c0_seq1
RPKM
X.1
X.2
X.3
